# Quantitative *in silico* Analysis of Neurotransmitter Pathways Under Steady State Conditions

**DOI:** 10.3389/fendo.2013.00137

**Published:** 2013-10-08

**Authors:** Daniela Calvetti, Erkki Somersalo

**Affiliations:** ^1^Department of Mathematics, Applied Mathematics and Statistics, Case Western Reserve University, Cleveland, OH, USA

**Keywords:** GABAergic, GABA cycling, flux balance analysis, Markov chain Monte Carlo, nitrogen metabolism

## Abstract

The modeling of glutamate/GABA-glutamine cycling in the brain tissue involving astrocytes, glutamatergic and GABAergic neurons leads to a complex compartmentalized metabolic network that comprises neurotransmitter synthesis, shuttling, and degradation. Without advanced computational tools, it is difficult to quantitatively track possible scenarios and identify viable ones. In this article, we follow a sampling-based computational paradigm to analyze the biochemical network in a multi-compartment system modeling astrocytes, glutamatergic, and GABAergic neurons, and address some questions about the details of transmitter cycling, with particular emphasis on the ammonia shuttling between astrocytes and neurons, and the synthesis of transmitter GABA. More specifically, we consider the joint action of the alanine-lactate shuttle, the branched chain amino acid shuttle, and the glutamine-glutamate cycle, as well as the role of glutamate dehydrogenase (GDH) activity. When imposing a minimal amount of bound constraints on reaction and transport fluxes, a preferred stoichiometric steady state equilibrium requires an unrealistically high reductive GDH activity in neurons, indicating the need for additional bound constants which were included in subsequent computer simulations. The statistical flux balance analysis also suggests a stoichiometrically viable role for leucine transport as an alternative to glutamine for replenishing the glutamate pool in neurons.

## 1 Introduction

In the glutamate/GABA-glutamine cycle of neurotransmission, where the ammonium fixation is essential in the synthesis of glutamine in astrocyte, a natural question still waiting for a definitive answer is how the ammonium pool in astrocyte is replenished (1, 2). The tight coupling between reactions and transports involved in neurotransmitter synthesis and cycling within the complex metabolic network, and the distribution of the functions into different compartments makes it hard, if not impossible, to manually follow the fate of the metabolites and to identify in quantitative terms stoichiometrically feasible steady states. The computational challenge, hampering standard optimization schemes, is rooted in the indeterminacy of the governing system of mass balance equations, which allows a continuum of possible solutions, and is made worse by the need of introducing bounds on some of the flux rates, for example imposing positivity to exclude solutions that are either thermodynamically impossible or would require physiologically unfeasible conditions. The statistical sampling approach provides one viable approach for the *in silico* study of complex metabolic networks (3–7).

In order to shed some light on the nitrogen metabolism and shuttling of amino groups between astrocytes and neurons during inhibition, we propose a complex, eight compartment metabolic model which comprises astrocytes, glutamatergic, and GABAergic neurons, each equipped with separate cytosol and mitochondria. We perform a statistical flux balance analysis of the metabolic pathway during inhibitory activity and verify whether the amino group shuttling mechanisms proposed in the literature are supported by the results of our computational simulations.

The focus of the present *in silico* study is on the synthesis and cycling of the inhibitory neurotransmitter GABA, with the specific aim of elucidating the source and fate of amino groups and ammonia during the GABA cycle. Among the different reaction capable of generating ammonia in the brain, phosphate-activated glutaminase, predominantly expressed in neurons, is considered the major source of cerebral endogenous ammonia (1). Various mechanisms have been proposed for transporting ammonia from neurons, where it is released, to astrocytes, where it is needed for the glutamine synthesis, including a diffusive process of ammonia, the alanine-lactate shuttle, and the branched chain amino acid shuttle (1, 2). Although the basic mechanisms of the shuttles are well understood, by considering the joint action of them rather than each one of them separately and isolated from others, a different picture of their role may emerge.

## 2 The Pathway Model

The template for the metabolic models that we test in this article is an eight compartment model, developed on the basis of the model in Calvetti and Somersalo (8), comprising separate cytosol and mitochondrial compartments for astrocytes, glutamatergic and GABAergic neurons, as well as blood and extracellular space (ECS) compartments, the latter one accounting also for the synaptic cleft, which in some models constitutes a separate compartment (9).

Each cell compartment is equipped with detailed cytosolic glycolytic pathway, including the reversible lactate dehydrogenase (LDH), and mitochondrial tricarboxylic acid (TCA) cycle and oxidative phosphorylation (OxPhos).

The complete malate-aspartate shuttle (MAS) included in each cell consists of the oxoglutarate carrier (OGC) exchanging malate (Mal) and α-ketoglutarate (AKG), and the aspartate-glutamate carrier (AGC1). To complete the shuttle, cytosol and mitochondria are equipped with aspartate aminotransferases (cAAT and mAAT, respectively), as well as with reversible malate dehydrogenases, converting oxaloacetate (OAA) to malate in cytosol (cMDH) or vice versa in mitochondria (mMDH).

Both glutamatergic and GABAergic neurons are equipped with mitochondrial malic enzyme (mME) (10), while in astrocyte malic enzyme is located in cytosol (cME) (11). Mitochondrial pyruvate carboxylase (PC) is included in astrocytes only (12).

Cytosolic glutamate (Glu) is packed in vesicles by presynaptic glutamatergic neurons and released to the synaptic cleft, from where it is quickly taken up by astrocytes. The astrocyte-specific enzyme glutamine synthetase (GS) located in the cytosol of astrocyte (13) catalyzes the amidation of glutamate to glutamine (14). The basic glutamate-glutamine cycle is completed by phosphate-activated glutaminase (PAG) which is believed to be located in the mitochondrial intermembrane (15). Since PAG replenishes the cytosolic glutamate pool, we include it in our model as a cytosolic enzyme in both glutamatergic and GABAergic neuron.

The inhibitory neurotransmitter γ-aminobutyric acid (GABA) is released by the GABAergic neurons into the synaptic cleft, and further taken up by the astrocytes. The GABA synthesis is catalyzed by the enzyme glutamic acid decarboxylase (GAD). This is a cytosolic enzyme with different isoforms (16) that are not distinguished in our analysis, and assumed to be present both in astrocytes and neurons.

Besides the neurotransmission, both glutamate and GABA play a role in the cell metabolism, thus complicating the tracking of amino groups in the network to determine their fate. GABA can enter the mitochondria through a mechanism not completely understood (17). In our model, the GABA permeation is assumed to take place through a GABA carrier, although a GABA/glutamate antiporter mechanism has been suggested (18), and recently identified and described in cell membranes of prokaryotes (19). After entering mitochondria, GABA can be transaminated by the enzyme GABA transaminase (GABA-T) to succinate semialdehyde (SSA), and subsequently converted by succinate semialdehyde dehydrogenase (SSADH) to succinate (Suc) (20). These reactions constitute the GABA shunt, providing an alternative route from α-ketoglutarate to succinate bypassing some TCA reactions. By GABA-T, α-ketoglutarate forms glutamate, which can be further transaminated by mAAT, transported into cytosol, or alternatively oxidized to α-ketoglutarate by the enzyme glutamate dehydrogenase (GDH), which facilitates a bidirectional reaction; however, at the physiological ammonium concentrations and redox levels, the oxidative direction of the reaction is strongly favored (1, 21).

In addition to the AAT, glutamate may participate in other transaminase processes. We include in our model the bidirectional cytosolic alanine transaminase (ALT), in which alanine (Ala) is transaminated to pyruvate, while α-ketoglutarate forms glutamate. This reaction, together with LDH, constitutes the lactate-alanine cycle (22, 23) proposed as a carrier of the ammonium (${\text{NH}}_{4}^{+}$) between astrocytes and neurons.

Another important shuttle of the amino group identified in the brain tissue is the branched chain amino-transferase (BCAT), which is a cytosolic enzyme (BCATc) in neurons and mitochondrial (BCATm) in astrocyte (24). For simplicity, we use leucine (Leu) as a common representative of the three branched chain amino acids (BCAA) (leucine, isoleucine, and valine), and α-ketoisocaproate (KIC) as the representative of the corresponding branched chain α-ketoacids (BCKA). In mitochondria, α-ketoisocaproate can be oxidized to acetyl-coenzyme-A (ACoA), although preferably reaminated back to BCAA (25). The BCAAs have been suggested to constitute an important alternative for shuttling ammonia between astrocyte and neuron.

As pointed out above, our model allows the passage of GABA through the mitochondrial membrane by a mechanism that has not been identified in mammalian brain. Cytosolic glutamate, on the other hand, has access to mitochondria through the ACG1 exchanger. Similarly, we include in our model a glutamate-hydroxyl carrier (GC) that allows the passage of glutamate through the mitochondrial membrane without tight coupling with aspartate (20). In addition, we allow the passage of malate through the mitochondrial membrane by the dicarboxyl carrier (26). Likewise, we assume a transport mechanism for branched chain amino acids and α-ketoisocaproate across the astrocytic mitochondrial membrane.

In our model, the three cell types can uptake glucose (Glc) from the ECS compartment, and exchange oxygen (O_2_), carbon dioxide (CO_2_), ammonium, and lactate (Lac). Following Cooper (1), we do not distinguish between ammonium and ammonia, although a detailed modeling of the diffusion through the membranes would deserve more attention. We also assume a mechanism for passing branched chain amino acids, α-ketoisocaproate and alanine between the cells and ECS. In contrast to our earlier models, aspartate is not included as an exchangeable substance because it is not believed to be an appropriate shuttle between the cells due to its excitatory nature (27, 28).

The substances exchanged between blood and ECS compartments include glucose, lactate, oxygen, carbon dioxide, ammonium, alanine, leucine, and glutamine. Although there is evidence of the presence of glutamate transporters at the blood-brain-barrier (BBB) (29), we assume here that the clearance by astrocytes is fast enough for the glutamate transport to be considered insignificant.

The energetic cost of neural activity is hard to estimate purely on a stoichiometric basis: The energetic needs of the cycling of the neurotransmitters constitute only a part of the total cost that includes the membrane potential homeostasis, signal propagation, and vesicle formation. In Attwell and Laughlin (30), a careful stoichiometric analysis of the energetic need of glutamate cycling between neurons and astrocytes leads to an estimate of approximately three ATP per each glutamate molecule. However, the authors point out that the total energetic need is higher. The energetic needs of astrocytes is even less well known. Part of the difficulty of incorporating a stoichiometrically justified figure for the energetic cost stems from the lumped nature of the model which needs to integrate non-local ion translocation processes in a well-mixed compartment representation.

A semi-empirical approach has been suggested in the previous works of the authors (31, 32), and we will be adopt a similar approach in this paper. In Sibson et al. (33) it was empirically shown that in rat brain, the cerebral metabolic rate (CMR) of glucose in neurons is in almost 1:1 proportion to the glutamate flux, an observation that has been corroborated by several groups. A refined analysis was given by Hyder et al. (34), estimating that the total neurotransmitter cycle flux of glutamate is 68% of the total neuronal oxidative CMR of glucose, while the total neurotransmitter cycle flux of GABA is 21% of the total neuronal oxidative CMR of glucose. Estimating that each completely oxidized molecule of glucose produces 30–38 ATP molecules, depending on details included, we may conclude that the total cost for maintaining each unit of glutamate flux corresponds to 21–26 units of ATP converted to ADP + Pi (no GABAergic activity), and 6–8 units of ATP for maintaining one unit of GABA flux (no glutamatergic activity). As demonstrated in Calvetti and Somersalo (8), in spite of its coarseness, in simulations these flux estimates yield an energetic cost that correspond well to the data reported in Sibson et al. (33).

For simplicity, we include in our model a virtual vesicular compartment in the cytosol of glutamatergic and GABAergic neurons, and attach the total energetic cost in the flux of packing the neurotransmitters in the vesicular form, see Table 1 for details.

**Table 1:** **List of reactions included in the metabolic network model**.

	Reaction	cA	mA	cGlt	mGlt	cGA	mGA
Glycolysis	GLC + ATP → G6P + ADP	×		×		×	
	G6P + ATP → 2GA3P + ADP	×		×		×	
	GA3P + Pi + NAD^+^ → BPG + NADH	×		×		×	
	BPG + 2ADP → Pyr + 2ATP	×		×		×	
LDH	Pyr + NADH ⇌ Lac + NAD^+^	×		×		×	
ALT	Pyr + Glu ⇌ Ala + AKG	×		×		×	
ATP-H	ATP → ADP + Pi	×		×		×	
GAD	Glu → GABA + CO_2_	×		×		×	
PAG	Gln → Glu + ${\text{NH}}_{4}^{+}$			×		×	
MDH	OAA + NADH ⇌ Mal + NAD^+^	×		×		×	
AAT	Asp + AKG ⇌ OAA + Glu	×	×	×	×	×	×
ME	Pyr + CO_2_ + NADH ⇌ Mal + NAD^+^	×			×		×
GS	Glu + ${\text{NH}}_{4}^{+}$ + ATP → Gln + ADP + Pi	×					
GDH	Glu + NAD^+^ ⇌ AKG + ${\text{NH}}_{4}^{+}$ + NADH		×		×		×
PDH	Pyr + CoA + NAD^+^ → ACoA + NADH + CO_2_		×		×		×
PC	Pyr + CO_2_ + ATP ⇌ OAA + ADP + Pi		×				
TCA cycle	ACoA + OAA → CIT + CoA		×		×		×
	CIT + NAD^+^ → AKG + CO_2_ + NADH		×		×		×
	AKG + CoA + NAD^+^ → SCoA + CO_2_ + NADH		×		×		×
	SCoA + ADP + Pi → Suc + CoA + ATP		×		×		×
	Suc + NAD^+^ → Fum + NADH		×		×		×
	Fum → Mal		×		×		×
	Mal + NAD^+^ → OAA + NADH		×		×		×
GABA-T	GABA + AKG → SSA + Glu		×		×		×
SSADH	SSA + NAD^+^ → Suc + NADH		×		×		×
BCAT	Leu + AKG ⇌ KIC + Glu		×	×		×	
BCKDH	KIC + CoA → ACoA + CO_2_		×		×		×
OxPhos	O_2_ + 6ADP + 6Pi + 2NADH → 6ATP + 2NAD^+^		×		×		×

	**Virtual reactions with energetic cost**						

	Glu + 25ATP → Glu_vesicular_ + 25ADP + 25Pi			×			
	GABA + 8ATP → GABA_vesicular_ + 8ADP + 8Pi					×	

The symbol “ →” is used for irreversible reactions, while “⇌” is used for reversible ones. The relevant abbreviated enzyme acronyms referring to the corresponding reactions are indicated in the first column. The columns refer to compartments that are the locus of the enzyme: cA/mA, cytosol/mitochondria of astrocyte; cGlt/mGlt, cytosol/mitochondria of glutamatergic neuron; cGA/mGA, cytosol/mitochondria of GABAergic neuron. The virtual reactions, transporting glutamate and GABA to a virtual vesicular compartment for neurotransmission are included to attach an undifferentiated energetic cost for the total neurotransmitter cycling, see text for justification.

The energetic cost of astrocytic neurotransmitter recycling is only partly known: the cost of glutamine synthetase and Na+/H+ extrusion after ion co-transport with glutamate is assessed at about 2ATP/glutamate in Attwell and Laughlin (30). In the present paper we use this stoichiometry for GABA recycling, understanding that it may underestimate the total energetic cost for astrocyte functions. In fact, experimental data suggest that astrocytes use from 15% to more than 25% of brain energy (35–37) for various functions, including K^+^ clean-up and calcium signaling, neither of which is explicitly accounted for in the model. To counterbalance the underestimate, our model enables the cells to use energy for unspecified activities by including an ATP hydrolysis in each cell. As pointed out in Calvetti and Somersalo (8), the activity level of this flux, in particular in astrocytes, can be significantly elevated. Details will be given later when the computed examples are described.

A complete list of the reactions included in the model is given in Table 1. The configuration described by our model refers to well-mixed compartments in which the input variables are specified target levels of the cerebral metabolic rates of glucose and lactate, and the brain activity is simulated by specifying, in terms of target flux values, the efflux of the pertinent neurotransmitter, which in the present investigation is GABA. Table 2 lists the transport fluxes of selected species between some of the compartments.

**Table 2:** **The transports implemented in the model**.

	Blood		ECS		Cytosol		Mitochondria
Glc	●	→	●	→	●		●
Lac	●	↔	●	↔	●		●
Pyr	●		●		●	→	●
O_2_	●	→	●	→	●	→	●
CO_2_	●	←	●	←	●	←	●
${\text{NH}}_{4}^{+}$	●	→	●	↔	●	↔	●
Leu	●	→	●	↔	●	↔	●
KIC	●		●	↔	●	↔	●
Ala	●	↔	●	↔	●		●
ATP	●		●		●	↔	●
ADP	●		●		●	↔	●
Pi	●		●		●	↔	●
Glu	●		●	↔	●	↔	●
GABA	●		●	↔	●	↔	●
Gln	●	←	●	↔	●	↔	●
	Malate-aspartate shuttle (MAS)						
OGC	Mal (cyto) + AKG (mito) → Mal (mito) + AKG (cyto)						
AGC1	Glu (cyto) + Asp (mito) → Glu (mito) + Asp (cyto)						

In the columns, “ →” and “ ←” mean that the transport has been implemented as unidirectional in the direction indicated by the arrow, while “ ↔” means that *a priori* no preferred direction is specified. Observe that in the numerical simulations, we may implement bound constraints that limit the direction of some of the reactions and transports, as indicated in the text.

## 3 Computational Approach

Under steady state hypotheses, the metabolic model is characterized by reaction fluxes and transport rates that must be in stoichiometric equilibrium. Each reaction *R_i_* within a specific compartment is assigned a reaction flux φ*_i_*. Similarly, if a substrate is exchanged between two adjacent compartments through a transport *T_i_*, the latter is assigned a transport rate *j_i_*, expressed in the same units as the reaction fluxes. The reaction fluxes and transport rates are then collected in an array, the flux-transport vector *u*,

(1) $$u=\left[\begin{array}{c} \varphi \\ j \end{array}\right],\;\mathrm{with}\;\varphi =\left[\begin{array}{c} \varphi_{1}\\ \vdots \\ \varphi_{n} \end{array}\right],\;j=\left[\begin{array}{c} j_{1}\\ \vdots \\ {\;j}_{k} \end{array}\right],$$

where *n* and *k* are the total number of reactions and transports in the system.

Given a transport *T_i_* or a reaction *R_i_*, we define the stoichiometric vector *s^i^* by specifying the number of units of each biochemical species in each compartment which are either produced or depleted if the transport or reaction runs alone for one time unit. Thus, the length of each stoichiometric vector equals the total number of species in the compartment model. Observe that a metabolite appearing in several compartments is counted as a different species in each compartment. After arranging the stoichiometric vectors as the columns of the stoichiometric matrix,

(2) $$A=\left[\begin{array}{cccc} s^{1} & s^{2} & \cdots & s^{n+k} \end{array}\right],$$

the steady state equilibrium condition for the flux-transport vector can be expressed in the form

(3) Au=r.

Above, the vector *r* describes the transport rates of substances to and from outside the compartment model: its entries are all zeros for compartments that are not communicating with the outside world through convection or diffusion, while for the species exchanged through BBB and the blood flow, they represent the CMRs of the tissue sample that the model is describing. For further details, see Table 3.

**Table 3:** **Input values defining the non-vanishing components of the right hand side *r* in equation (3)**.

*r_j_* = *Q*/*F*$\left(C_{j}^{a}-C_{j}^{v}\right)$	*Q* = 0.55	*F* = 1	
	$C_{j}^{a}-C_{j}^{v}$	$C_{j}^{a}$	$C_{j}^{v}$
Glc	0.54 ± 0.05	–	–
Lac	−0.18 ± 0.02	–	–
O_2_ (free and bound)	–	9.15 ± 0.5	Estimated
CO_2_ (free and bound)	–	23 ± 1	Estimated
	0 ± 0.2	–	–
Leu	0 ± 0.1	–	–
Gln	0 ± 0.1	–	–

The system is scaled so that it corresponds to 1 g tissue. The units of the blood flow *Q* are milliliter per minute, the mixing ratio *F* is unit free, and the concentrations are given in micromoles per milliliter. The flux values are expressed in micromoles per minute. In the table, “estimated” means that the quantity is included in the list of unknowns and estimated together with the flux values. Observe that the formula for *r_j_* above for oxygen and carbon dioxide refers to concentrations of free gases, and a conversion from bound to free needs to be performed [see, e.g., Ref. (51)].

The reaction fluxes and transport rates are subject to bound constraints: a flux φ*_i_* of a reaction *R_i_* that is thermodynamically possible only in one direction must respect the positivity condition φ*_i_* > 0. Following the Bayesian paradigm, some of the positivity bounds may be purely *a priori* bounds, meaning that a positivity constraint may be implemented if there is a good reason to believe that a net flux of a bidirectional reaction or transport has a preferred direction, such as oxygen entering rather than exiting the tissue (38). Further, we may assume that all the reaction fluxes and transport rates must be bounded by some, possibly large, upper bound *V*_max_. The system of linear constraints is expressed in matrix form as

(4) Cu≥c,

where *c* is a vector with as many entries as we have the inequality constraints, the matrix *C* contains the coefficients of the linear expressions in the inequalities, and the matrix inequality is assumed to hold component-wise.

Methods for finding a feasible flux-transport vector satisfying equation (3) with bounds equation (4) are discussed in the classical flux balance analysis (FBA) literature, see, e.g., Kauffman et al. (39). A well-known problem in FBA is the lack of a unique solution, i.e., the stoichiometry and bounds alone are not sufficient to identify a unique steady state. It is this non-uniqueness which gives the system the flexibility to adjust to changing physiological conditions. Rather than forcing the uniqueness of the solution by adding extra conditions, a task which may require constructing artificial objectives for the system, we seek to explore computationally the full set of feasible steady state configurations. The methodology to achieve this, which is based on a probabilistic description of the problem, has been developed in a series of papers by the authors, see, e.g., Heino et al. (7); Calvetti and Somersalo (8); Calvetti et al. (9), and references therein. We give a concise overview of the approach below.

In this probabilistic setting, feasible flux-transport vectors are assumed to be distributed in the space of all vectors so as to approximately satisfy both the equilibrium condition equation (3) while strictly respecting the bound constraint inequality (4). This is achieved by defining a truncated Gaussian probability density

(5) $$\pi \left(u\right)\propto H\left(Cu-c\right)\;\exp\;\left(-\frac{1}{2}{\left(Au-r\right)}^{T}\Sigma^{-1}\left(Au-r\right)\right).$$

Here, “ ∝” stands for “proportional to,” *H* is the multidimensional Heaviside function that vanishes except when all the components of the vector *Cu* − *c* are positive, in which case it assumes the value one, and Σ is a covariance matrix, which we assume to be diagonal. The diagonal entries of Σ express how tightly each one of the steady state equations is enforced. We refer to the square roots of these entries as standard deviations of the model equations. A rather stringent condition is required in our calculations: the standard deviation is set to σ = 0.005 μmol/min for every model equation, except for equations that define the influx/efflux from blood to ECS. The latter standard deviations are given in Table 3.

To investigate feasible steady state configurations with some of the fluxes close to a desired target value, the distribution can be modified accordingly in a simple way: the belief that a given flux or transport *u_ℓ_*, such as the efflux of a given neurotransmitter, should be close to a target value $u_{\ell }^{\mathrm{target}},$ can be implemented by augmenting the probability density equation (5) with an extra factor,

(6) $$\pi \left(u\right)\to \pi \left(u\right)\times \exp\;\left(-\frac{1}{2w^{2}}{\left(u_{\ell }-u_{\ell }^{\mathrm{target}}\right)}^{2}\right),$$

where *w* > 0 is the standard deviation controlling how stringently the target value is pursued. We specify the values of these parameters later on. To explore the resulting probability density, and thus the distribution of all feasible flux-transport configurations, we use Markov chain Monte Carlo (MCMC) techniques to generate a representative sample of those vectors,

(7) $$S=\left\{u^{1},u^{2},\ldots ,u^{N}\right\},$$

such that for *N* large, the vectors *u^j^* are, at least asymptotically, distributed according to the probability density π(*u*). For details, see Heino et al. (7). Based on this sample, we obtain a summary statistics of the distribution, e.g., by computing the sample mean and variance. In the sequel, we shall use almost exclusively the sample mean as a representative flux-transport configuration for a given stoichiometric model with bounds and target controls defined in equations (4) and (6).

The values for the input parameters used in the computations are given in Table 3.

## 4 Quantitative Analysis of Ammonium Shuttling

**Figure 1:**
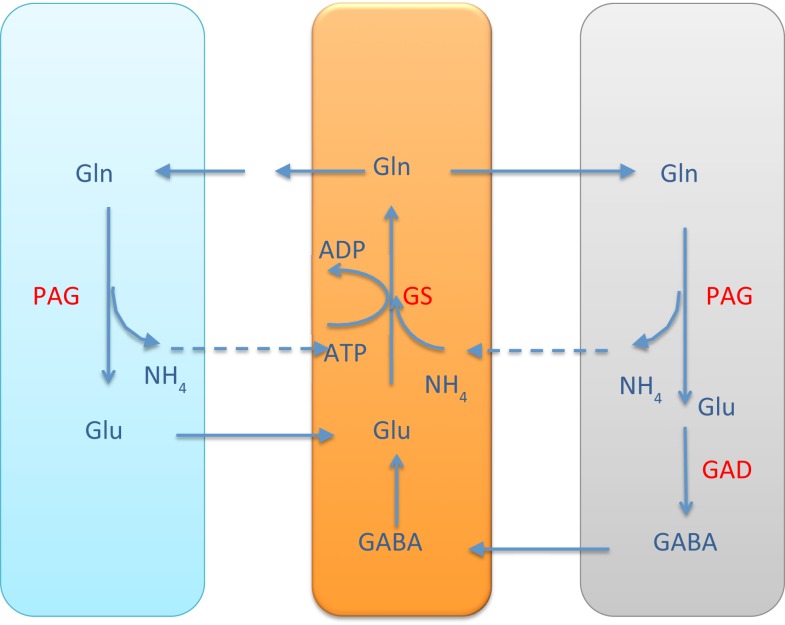
**The glutamate-glutamine cycle and GABA-glutamine cycles**. The excess ammonium freed by PAG in the neurons needs to be transported back to astrocyte, where a shortage is created by the GS activity. In the pure glutamate/GABA-glutamine cycle paradigm, the ammonium diffusion or surrogate shuttle mechanisms need to go at the flux rate of the glutamine efflux from astrocyte. The quantitative analysis indicates that the full picture may be more complex.

It is commonly accepted that the glutamate and GABA neurotransmitter cycling between neurons and astrocyte is completed by the glutamine transport, following the glutamate/glutamine cycle for excitatory transmission between the glutamatergic neuron and astrocyte, or the GABA/glutamine cycle for the inhibitory transmission between GABAergic neuron and astrocyte. Both cycles require the ammonium fixation by GS in astrocytic cytosol, and metabolizing glutamine into glutamate in neuron by PAG. In both transmitter cycles, a stoichiometric shortage of one ${\text{NH}}_{4}^{+}$ in the astrocytic cytosol, and excess of one ${\text{NH}}_{4}^{+}$ in the neuronal cytosol ensues, see Figure 1.

As pointed out in Rothman et al. (2), the stoichiometric imbalance requires one of the following alternatives to take place:

1. the excess ${\text{NH}}_{4}^{+}$ in the neuronal cytosol diffuses via ECS to the astrocyte, probably in the form of NH_3_, or
2. the excess ${\text{NH}}_{4}^{+}$ in the neuronal cytosol diffuses in the mitochondria and is fixed to α-ketoglutarate by GDH to form glutamate.

The former alternative readily resumes the stoichiometric equilibrium of ammonia, while the latter requires a further shuttling mechanism to restore the balance. In the literature, different mechanisms have been proposed.

**Figure 2:**
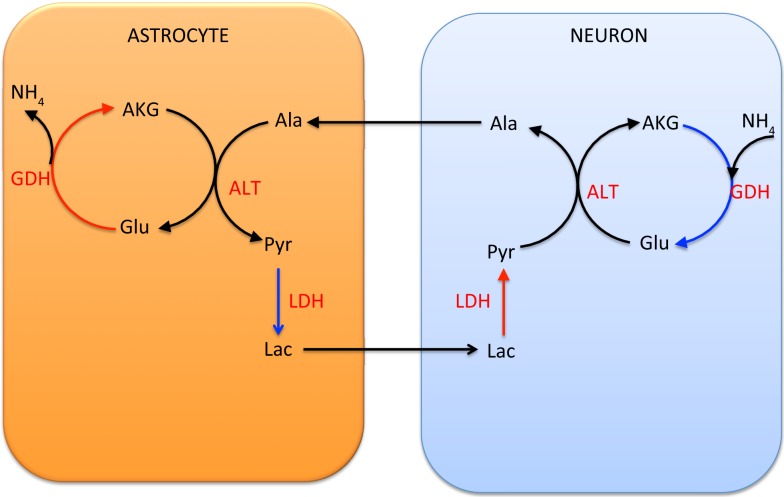
**The lactate-alanine cycle between astrocyte and neuron**. Observe that in order to complete the cycle, lactate needs to move from astrocyte to neuron. The shuttle is in redox equilibrium, since in both cells the oxidative (red arrows) and reductive (blue arrows) reactions compensate each other. However, in order for this shuttle to be efficient at transferring ammonium, it requires reductive activity of the GDH in the neuronal mitochondria, a scenario that has been questioned in the literature both for theoretical reasons and in the light of experimental evidence (1).

In the alanine-lactate shuttle (22), the mitochondrial glutamate in neuron enters cytosol either by the AGC1 exchanger or the GC carrier, and consequently is transaminated to α-ketoglutarate by ALT, while concurrently forming alanine from pyruvate. In this scenario, alanine is then shuttled to astrocyte, where it is transaminated to pyruvate by ALT, concomitantly forming glutamate from α-ketoglutarate. To attain the carbon balance, pyruvate is shuttled from astrocyte to neuron in the form of lactate, produced by LDH, which converts lactate to pyruvate in a reverse reaction in neuron. We refer to Figure 2 for an illustration.

**Figure 3:**
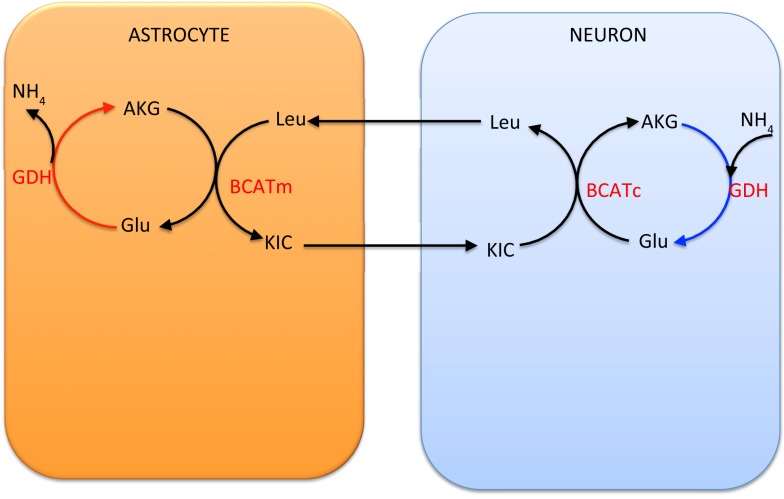
**The branched chain amino acid cycle**. In order to effectively move the ${\text{NH}}_{4}^{+}$ from neuron to astrocyte, the latter has to be fixed to α-ketoglutarate by mitochondrial GDH, thus requiring this reaction to go in the reductive direction, hence running into the same criticism as the lactate-alanine shuttle (see Figure 2).

An alternative shuttle mechanism uses the branched chain amino acids, leucine in particular in our model, as carriers of the amino group. In this scenario, glutamate is transaminated to α-ketoglutarate by BCATc in the cytosol of the neuron, while α-ketoisocaproate is converted into leucine. The branched chain amino acids and corresponding keto acids are then exchanged between the neuron and the astrocyte, and the reverse reaction, facilitated by BCATm, produces glutamate and α-ketoisocaproate in the mitochondria of the astrocyte. This shuttle mechanism is illustrated schematically in Figure 3.

Several factors complicate the analysis of the interplay between the various shuttling mechanism, including the following:

1. The different mechanisms are probably completing each other, none of them alone compensating the ammonium imbalance;
2. Glutamate has a complex role, being not only a neurotransmitter but also a metabolite;
3. The two proposed shuttling mechanisms require that GDH in neuron runs in the reductive direction with a significant flux, which has been experimentally and theoretically been questioned as a possible option;
4. both ${\text{NH}}_{4}^{+}$ and leucine are assumed to be replenished from the blood, while glutamine may leak out, making the shuttling mechanisms stoichiometrically leaky.

These are some of the considerations which make a flexible computational tool for following the fluxes particularly attractive.

## 5 Numerical Simulations

In Calvetti and Somersalo (8), three different simulated activation levels were considered: Excitation state, characterized by high glutamate efflux from glutamatergic neuron, awake state, defined by moderate glutamate efflux, and inhibition state, in which the GABA efflux from GABAergic neuron is set at a specified level. These states are not intended to model the whole brain, which is known to be predominantly glutamatergic (40, 41), but rather a small portion of it that is assumed to correspond to the prescribed activity. Without specifying the volume of the domain modeled, we scale the fluxes to correspond a volume of one gram tissue. Our *in silico* analysis focuses on inhibitory activity, which is achieved by defining a target value for the GABA efflux from the GABAergic neuron (nGABA). In equation (6), we choose

$$u_{\ell }=j_{\mathrm{nGABA}\to \mathrm{ECS}}^{\mathrm{GABA}},\;u_{\ell }^{\mathrm{target}}=0.13\;\;\mu \mathrm{mol}∕\min.$$

This is the level of inhibitory activity state used in Calvetti and Somersalo (8), based on the experimental analysis in Shulman et al. (42) and Hyder et al. (34). Here, only the inhibitory activation state is considered as a representative of complex interplay between the astrocytes and the two neuron types.

The parameter *w* in equation (6) defining the standard deviation of the GABA flux from the target value is set to *w* = 0.005 μmol/min. While it is understood here that the GABA efflux should be a response to some excitatory activity, we do not explicitly specify the level of excitatory activity in the model, but rather let it be determined by the stoichiometry. By the way the model is set up, the uptake of nutrients, oxygen, and glutamine of the glutamatergic neuron settle to some levels that satisfy the equilibrium conditions. In the various simulations with the current model discussed later, the mean glutamate efflux from glutamatergic neurons to ECS is 0.08–0.09 μmol/min. The energy demand of the astrocytes, measured in terms of mean ATP turnover, in all our simulations is approximately 45% of the total ATP turnover. This indicates a high oxidative activity of astrocyte during inhibition, which is in line with the earlier computational results (8) as well as with experimental findings (37, 43).

Using the software package Metabolica, we generate a sample of *N* = 200,000 sample vectors *u^n^*, each of them representing a possible equilibrium or near-equilibrium flux configuration. In the following analysis, we report for the most part the mean transport-flux vector that is obtained by averaging over the sample, that is, we define the mean $\bar{u}$ by

$$\bar{u}=\frac{1}{N}\sum_{n=1}^{N}u^{n}.$$

We run three different tests, differing from each other by the imposed positivity constraints specified below, designed to put the focus on ammonium traffic and GABA formation.

### 5.1 Oxidative GDH and ammonia diffusion

In the first computational simulation, we constrain the directions of some key important fluxes. To analyze the ammonium uptake pattern, we first assume that GDH in all cell types goes in the oxidative rather than reductive direction, that is,

(8) $$\begin{array}{l} \mathrm{GDH}:\mathrm{Glu}+{\mathrm{NAD}}^{+}\to \mathrm{AKG}+{\mathrm{NH}}_{4}^{+}+\mathrm{NADH},\;\varphi_{\mathrm{GDH}}\geq 0. \end{array}$$

These constraints constitute three rows in the system equation (4), one corresponding to each cell type.

In addition, we prescribe the direction of the branched chain amino acid transport from ECS to astrocyte (ast), by implementing the positivity constraint

(9) $$j_{\mathrm{ECS}\to \mathrm{ast}}^{\mathrm{Leu}}\geq 0,$$

constituting an additional row in the system equation (4). We point out that this constraint is an *a priori* bound, included to make the model conform with the proposed shuttle mechanism as well as with the observation that leucine is predominantly taken up by astrocytes rather than neurons (44, 45). The effect of removing the constraint is also investigated later on.

#### 5.1.1 Ammonium traffic through ECS

**Figure 4:**
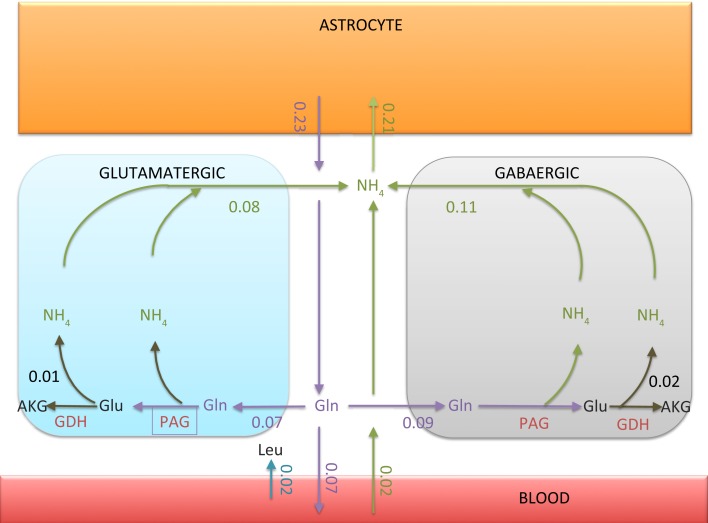
**The transport rates of glutamine and ammonium between ECS and other compartments**. The numbers indicated in the figure are in units micromoles per minute per 1 g tissue. Observe that the ammonium flux into the astrocyte is 0.02 μmol/min smaller than the glutamine efflux. The imbalance, however, is compensated by the influx of leucine from blood to ECS, as indicated in the figure. The net influx of ${\text{NH}}_{4}^{+}$ carried by leucine into astrocyte turns out to be exactly 0.02 μmol/min.

As expected, the bound constraint equation (8) disables the ammonium fixation in the neuron, and therefore makes both the alanine-lactate shuttle and the BCAA shuttle ineffective as possible pathways for trafficking ${\text{NH}}_{4}^{+}$ from neuron to astrocyte. Figure 4 summarizes quantitatively the ammonium trafficking between the compartments under the constraint equation (8).

There are two explanations why the ${\text{NH}}_{4}^{+}$ flux from ECS into the astrocyte is not equal to the glutamine efflux from astrocyte to ECS. First, the tissue takes up leucine from the blood, which in turn is taken up by astrocyte, thus providing a source of ammonium. Second, some of the glutamine synthesized in astrocyte is released in the blood and is thus removed from the system. We observe that the ${\text{NH}}_{4}^{+}$ flux out of the neurons is slightly higher than the glutamine flux into the neurons. The difference is due to the oxidation of glutamate in neurons, GDH oxidizing glutamate to α-ketoglutarate. The percentage of glutamine-derived glutamate which is oxidized is about 14% in glutamatergic neuron and 22% in GABAergic neuron. Also, there is a slight offset between the ammonium uptake flux of the astrocyte and the glutamine efflux. The difference, or missing ammonium in astrocyte, is compensated by the uptake of leucine from the blood.

#### 5.1.2 Glutamine as precursor of GABA

**Figure 5:**
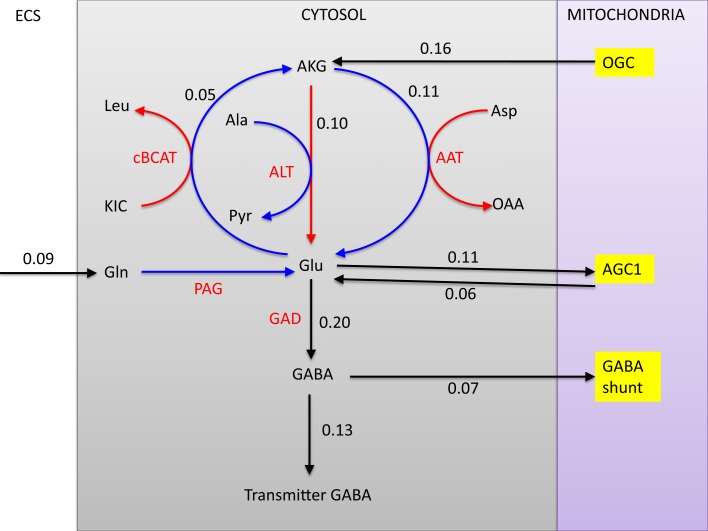
**The fates of glutamine in the GABAergic neuron**. All glutamine is transformed into glutamate in the cytosol by PAG, after which the pathway branches. The three transaminases with respective flux rates (cBCAT, ALT, and AAT) are marked in red. Observe that the rate of GAD is more than twice the rate of glutamine uptake and PAG. Of all GABA produced, 35% enters the mitochondria where it is transaminated by GABA-T and further oxidized. The glutamine influx comprises about 70% of the transmitter GABA efflux, although the complex pathway pattern complicates the tracking of the precursors.

The simplified picture of the GABA/glutamine cycle is that glutamine is taken up by GABAergic neuron, transformed to glutamate by PAG and further to GABA by GAD. Our analysis indicates that while this core chain is valid, the full picture is much more complicated. The mean fluxes indicate that the GAD activity (0.2 μmol/min) is more than twice the PAG (0.09 μmol/min) activity, raising the question of the origin of the extra glutamate in the cytosol of the GABAergic neuron. Mitochondrial glutamate can be replenished via three transaminases (AAT, ALT, and BCAA), and two transport mechanisms (AGC1 and glutamate-hydroxyl carrier GC) from the cytosol. As indicated in Figure 5, in a combined action, they exactly account for the excess glutamate. Another noteworthy detail is that the GAD produces not only the neurotransmitter GABA, but also a significant amount of GABA that is oxidized in the mitochondria, corresponding to about 35% of all GABA produced by GAD. The GABA entering mitochondria feeds the GABA shunt, the rate of which is slightly less than 50% of the total TCA cycle flux measured by the succinate dehydrogenase flux. This result is in line with the experimental results in rat brain reported in Hassel et al. (46).

### 5.2 Bidirectional GDH and the role of amino group shuttles

**Figure 6:**
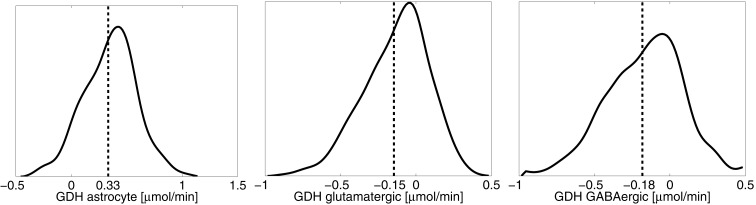
**The smoothed histograms of the GDH fluxes in each of the three cell types, calculated from the sample of 200,000 realizations**. The sample mean in each cell is indicated by the vertical line. Observe that the sum of the mean activities in the neurons equal the negative of the astrocytic activity.

The results reported in the previous section demonstrate quite clearly that without neuronal GDH running in the reductive direction, both the lactate-alanine shuttle and the branched chain amino acid shuttle play only a marginal role in the ammonium group traffic. This observation underlines the difficulty of reconciling the proposed shuttle mechanisms with experimental observations that brain tissue is incapable of ammonium fixing even under hyperammonemic conditions (21, 47, 48). To test computationally the viability and mutual equilibrium values of these shuttles, we repeat the sampling, removing the constraint inequality (8) from all cell types, which is tantamount to allowing bidirectional GDH activity. What we anticipate in this case is a rather significant reductive GDH activity in both neuron types. Figure 6 shows the smoothed histograms of the GDH activity in each cell type, indicating also the sample mean. The histograms indicate that a wide range of GDH activity levels are possible. However, in equilibrium conditions and with no bound constraints for the GDH, the fluxes seek to balance the reductive activity in neurons (φ_GDH_ < 0) by the oxidative activity in astrocyte (φ_GDH_ > 0). Because this equilibrium is not possible when the direction of the activity is restricted, the mismatch was balanced with ammonium diffusion between the cells.

**Figure 7:**
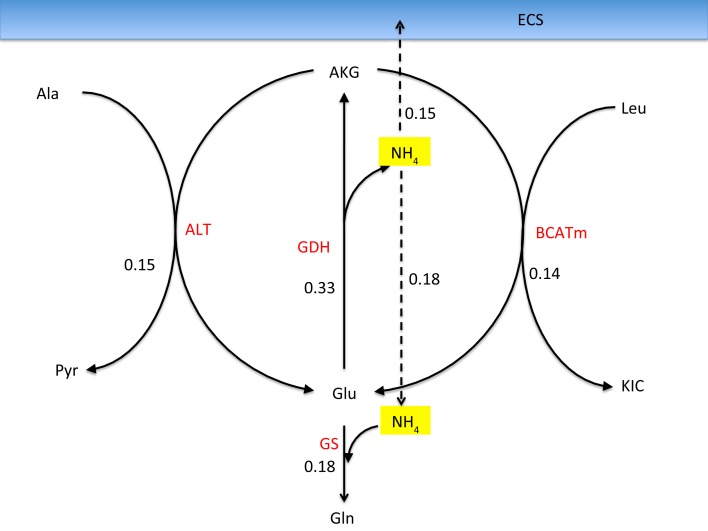
**Reactions in the astrocyte that liberate ammonium from alanine and leucine transported into astrocyte from the neurons when allowing a bidirectional GDH activity**. The values refer to the mean fluxes over a 200,000 sample. The oxidative GDH activity is higher than the GS flux, leading to an excess of ${\text{NH}}_{4}^{+}$ which is transported to the ECS and further taken up by neurons. This mean flux configuration may not be physiologically meaningful because of the unexpected efflux of ${\text{NH}}_{4}^{+}$ from astrocyte.

When GDH is assumed to be able to operate in both directions, it is possible to reduce, or even reverse the ${\text{NH}}_{4}^{+}$ diffusion from neuron to astrocyte by replacing it with the two shuttle mechanisms discussed in Section 4. The schematics in Figure 7 illustrate the mechanism in astrocyte that removes the ammonium from alanine and leucine via a joint action of GDH and the transaminases ALT and BCATm. The mean flux values in the figure indicate that the two shuttle mechanisms have almost the same level of activity. Moreover, the rate of the ${\text{NH}}_{4}^{+}$ production by this process is almost twice as large as the flux of GS depleting it: the residual ${\text{NH}}_{4}^{+}$ is released to ECS, from where it is taken up by the neurons. Therefore, the combined action of the two shuttle mechanisms, in the mean flux configuration, not only replaces the need for ammonium diffusion, but in fact, overwhelms it. This finding suggests that the mean flux configuration with freely reversible GDH in the neurons may not represent a physiologically meaningful steady state.

**Figure 8:**
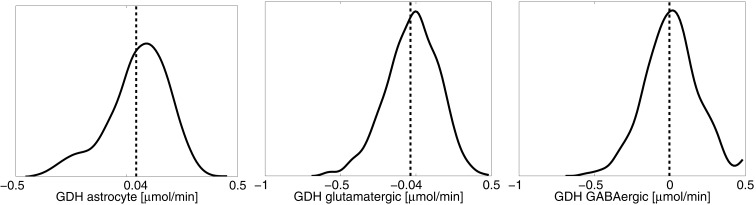
**The smoothed histograms of the GDH fluxes in each of the three cell types, calculated from the subsample of the flux vectors in which the ${\text{NH}}_{4}^{+}$ flux goes in the direction ECS → astrocyte**. The sample mean in each cell is again indicated by the vertical line.

**Figure 9:**
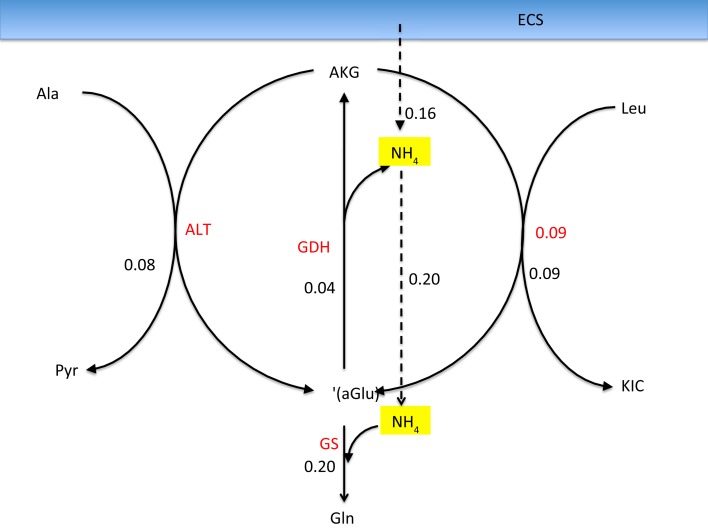
**The corresponding fluxes as in Figure 7, calculated as a mean over a subsample of those sample vectors for which the ${\text{NH}}_{4}^{+}$ flux is from ECS into the astrocyte**. The ammonia influx and the GDH flux add up to equal the GS flux. Observe that the GDH runs at a much lower rate than the two transaminases, and the corresponding lactate-alanine shuttle and the branched chain amino acid shuttle, demonstrating that considering the isolated shuttle mechanisms may be misleading.

One of the attractive features of the sampling-based approach to metabolic networks is that from the full sample of steady state configurations, it is possible to select subsamples of states that satisfy physiologically more meaningful bounds. In the present case, we select only those reaction-transport vectors *u^j^* for which the net ammonium flux from ECS into the cytosol of astrocyte is positive. Restricting the analysis to this subsample, we recompute the mean fluxes. Figure 8 shows the smoothed histograms of the subsampled GDH fluxes in each cell type. Interestingly, the mean GDH activity in the GABAergic neurons vanishes, while in the astrocyte and in the glutamatergic neurons GDH runs, in the mean, at the same low rate but in opposite direction. The counterpart of Figure 7 with mean fluxes calculated from the subsample are shown in Figure 9. The numbers indicate that about one fifth of the ammonium required by the GS originates from the GDH, while the rest enters the astrocyte by diffusion from the ECS.

### 5.3 Branched chain amino acid shuttle: An alternative for glutamine cycle?

**Figure 10:**
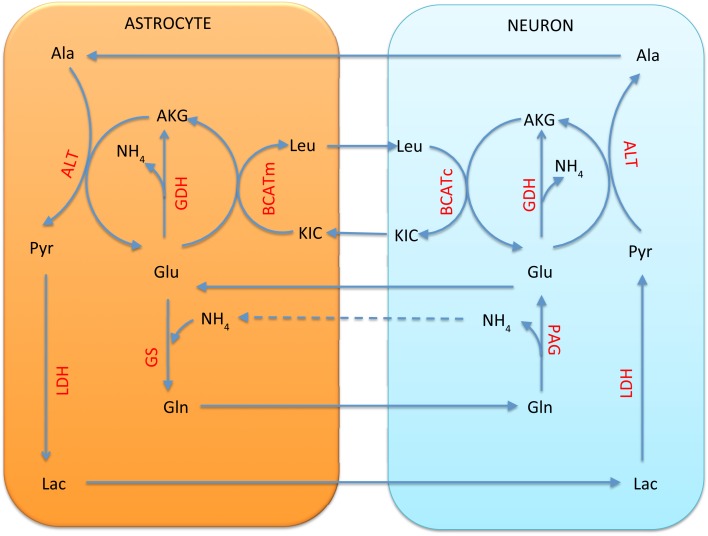
**The branched chain amino acid cycle can provide a stoichiometrically viable alternative for the traditional glutamate-glutamine cycle**. Leucine is transported from astrocyte to neuron, where it is transaminated to α-ketoisocaproate, forming glutamate from α-ketoglutarate. Unlike the glutamate-glutamine cycle, this cycle requires no return traffic of ammonia. A stoichiometric equilibrium can be found in which the GDH runs in oxidative direction in all cells, and the combined action of alanine-lactate shuttle and GDH replenishes the ${\text{NH}}_{4}^{+}$ pool in astrocyte. A flux of ${\text{NH}}_{4}^{+}$ from neuron to astrocyte is still needed for equilibrium.

The BCAA shuttle, which has been suggested as a vehicle for returning the ammonia freed by PAG in neuron into the astrocyte for fixation by GS, requires the questionable reductive action of GDH in neuron. When we remove the bound constraint inequality (9) on the direction of the transport of leucine, the pathway analysis tool Metabolica suggests an alternative function for BCAA that cannot be excluded by stoichiometric considerations alone. A stoichiometric equilibrium can be found in which BCAA is running in the reverse direction, transaminating astrocytic α-ketoisocaproate to leucine, which is transported to neuron, where the reverse reaction (Leu + AKG → KIC + Glu) replenishes the glutamate pool. In other words, leucine assumes the role of glutamine as a precursor of transmitter glutamate and GABA. This shuttle does not completely replace the glutamine as a precursor of glutamate in the mean equilibrium state, but acts as an additional source. The analysis suggests also that, when leucine can be taken up by neuron, the alanine-lactate shuttle runs in the direction suggested in Waagepetersen et al. (22), as shown in Figure 10. The situation is similar for the astrocyte and GABAergic neuron pair, in which the action of GAD from glutamate to GABA needs to be incorporated.

## 6 Conclusion and Perspectives

In this work, we investigate the neurotransmitter cycling in a steady state multi-compartment model using the computational tool Metabolica, with a particular emphasis on the traditional glutamate/GABA-glutamine cycling scenario between astrocytes and neurons. The basic brain metabolism multi-compartment model is enriched with other cycling mechanisms, such as branched chain amino acid shuttle and alanine-pyruvate shuttle. The focus in this work is on the stoichiometric implications of selected bound constraints on some of the key reaction fluxes and transport rates. This work is bridging the earlier computational works on brain energy metabolism by the authors with recently published works on neurotransmitter synthesis and cycling, [see Ref. (2, 49)]. As pointed out in Sibson et al. (33) and several other studies later on, the neurotransmitter cycling activity and the energy metabolism are tightly coupled, and therefore any model for one has implications for the other. However, the modeling paradigm adopted here which is based on modeling the reaction and transport fluxes in terms of distributions indicate that within the same energetic level, measured in terms of tissue glucose uptake, it is possible to find a range of stoichiometrically viable scenarios that differ in the details about the shuttling of the amino groups between the various cell types. As demonstrated in the article, the sample based approach makes it possible to narrow down the distributions by means of subsampling, thus excluding realizations that represent steady state configurations that lead to model predictions which are in conflict with observations.

The main findings of the article may be summarized in few points: first, if the GDH is only allowed to run in the oxidative direction in the neurons, as the experimental data suggest, a significant diffusion of ${\text{NH}}_{4}^{+}$ from neuron to astrocyte is needed to compensate the ammonium imbalance due to the GS-PAG activity. If, however, the reductive action of GDH is allowed with no further restrictions, the model seeks an equilibrium in which the GDH overcompensates, and the diffusion of ${\text{NH}}_{4}^{+}$ is reversed, going from astrocytes to neurons. Since this scenario does not seem plausible in the light of what is known experimentally, we selected from the sample of possible equilibrium states those flux vectors in which the ammonium traffic goes from ECS into the astrocyte, leading to an equilibrium in which the GDH activity is significantly reduced, and the equilibrium state is more in line with experimental observations.

Finally, we point out that if the branched chain amino acid shuttle is not restricted to run in the direction suggested in the literature, with leucine entering astrocyte, a viable equilibrium can be found, in which leucine plays a role of glutamine, enriching the glutamate pool in neuron without the need of compensating ${\text{NH}}_{4}^{+}$ diffusion from neuron to astrocyte. In our model, this glutamate/GABA-leucine cycle does not replace the traditional glutamate/GABA-glutamine cycle, but completes it. Although experimental evidence supports the uptake of leucine predominantly by astrocytes rather than neurons, the stoichiometric viability of this shuttle may be of interest under some atypical conditions.

The role that advanced statistical computational models may have in the brain research is to point out scenarios and alternative patterns in the complex metabolic network that may not be evident, and may be hard to find by simple flux balance analysis. In this way, the analysis may turn out to be useful as a guideline for designing new experiments. As pointed out in Rowley et al. (50), the details of neurotransmitter and amino group cycling are particularly important since they provide potential targets for drug discovery to control, e.g., epileptic seizures, hepatic encephalopathy, various mental disorders, or progression of neurodegenerative diseases.

## Conflict of Interest Statement

The authors declare that the research was conducted in the absence of any commercial or financial relationships that could be construed as a potential conflict of interest.
